# Progressive alignment of crystals: reproducible and efficient assessment of crystal structure similarity

**DOI:** 10.1107/S1600576722009670

**Published:** 2022-11-21

**Authors:** Aaron J. Nessler, Okimasa Okada, Mitchell J. Hermon, Hiroomi Nagata, Michael J. Schnieders

**Affiliations:** a Computational Biomolecular Engineering Laboratory, University of Iowa, Iowa City, Iowa, USA; b Sohyaku. Innovative Research Division, Mitsubishi Tanabe Pharma Corporation, Japan; c CMC Modality Technology Laboratories, Production Technology and Supply Chain Management Division, Mitsubishi Tanabe Pharma Corporation, Japan; Tohoku University, Japan

**Keywords:** structure comparison, crystal packing, crystal structure prediction, radius of gyration, MPI parallelization

## Abstract

During *in silico* crystal structure prediction of organic molecules, millions of candidate structures are often generated. These candidates must be compared to remove duplicates prior to further analysis (*e.g.* optimization with electronic structure methods) and ultimately compared with structures determined experimentally. The agreement of predicted and experimental structures forms the basis of evaluating the results from the Cambridge Crystallographic Data Centre (CCDC) blind assessment of crystal structure prediction, which further motivates the pursuit of rigorous alignments. Evaluating crystal structure packings using coordinate root-mean-square deviation (RMSD) for *N* molecules (or *N* asymmetric units) in a reproducible manner requires metrics to describe the shape of the compared molecular clusters to account for alternative approaches used to prioritize selection of molecules. Described here is a flexible algorithm called *Progressive Alignment of Crystals* (*PAC*) to evaluate crystal packing similarity using coordinate RMSD and introducing the radius of gyration (*R*_g_) as a metric to quantify the shape of the superimposed clusters. It is shown that the absence of metrics to describe cluster shape adds ambiguity to the results of the CCDC blind assessments because it is not possible to determine whether the superposition algorithm has prioritized tightly packed molecular clusters (*i.e.* to minimize *R*_g_) or prioritized reduced RMSD (*i.e.* via possibly elongated clusters with relatively larger *R*_g_). For example, it is shown that when the *PAC* algorithm described here uses single linkage to prioritize molecules for inclusion in the superimposed clusters, the results are nearly identical to those calculated by the widely used program *COMPACK*. However, the lower *R*_g_ values obtained by the use of average linkage are favored for molecule prioritization because the resulting RMSDs more equally reflect the importance of packing along each dimension. It is shown that the *PAC* algorithm is faster than *COMPACK* when using a single process and its utility for biomolecular crystals is demonstrated. Finally, parallel scaling up to 64 processes in the open-source code *Force Field X* is presented.

## Abstract 2

Evaluating crystal structure packings using coordinate root-mean-square deviation (RMSD) for *N* molecules (or *N* asymmetric units) in a reproducible manner requires metrics to describe the shape of the compared molecular clusters to account for alternative approaches used to prioritize selection of molecules. Described here is a fast algorithm called *Progressive Alignment of Crystals* (*PAC*) to evaluate crystal packing similarity using coordinate RMSD and introducing the radius of gyration as a metric to quantify the shape of the superimposed clusters.

## 1. Introduction

Organic crystals have significance due to their role in causing diseases such as gout (Terkeltaub, 2010 ▸) (monosodium urate monohydrate) and kidney stones (Moe, 2006 ▸) (calcium oxalate), their potential use in the low-pressure storage of gases within crystalline metal–organic frameworks (James, 2003 ▸; Furukawa *et al.*, 2010 ▸), and their use in the oral delivery of pharmaceuticals (Blagden *et al.*, 2007 ▸) such as paracetamol (Haisa *et al.*, 1976 ▸, 1974 ▸) (acetaminophen) and acetyl­salicylic acid (Wheatley, 1964 ▸; Vishweshwar *et al.*, 2005 ▸) (aspirin). During the pharmaceutical formulation process, crystallization screens often discover more than one crystal packing arrangement (*i.e.* polymorphs) based on testing an array of experimental conditions (*e.g.* solvent, pH, salt, temperature and pressure). Each solid form has unique physical properties (*e.g.* density, thermodynamic stability, melting temperature and solubility) driven by both intramolecular conformation and intermolecular interactions. For this reason, each polymorph can be covered by a unique patent and, in the case of a pharmaceutical solid form, must be considered individually for US Food and Drug Administration (FDA) approval (Kapczynski *et al.*, 2012 ▸). Crystal structure prediction can be performed *in silico* to complement experimental polymorph screens and thereby reduce the risk of a previously unknown stable polymorph emerging (Leelananda & Lindert, 2016 ▸). A variety of computational methods have been used to predict crystal structures (Day, 2011 ▸; Reilly *et al.*, 2016 ▸; Burger *et al.*, 2018 ▸; Price, 2008 ▸, 2014 ▸; Price & Price, 2011 ▸; Karamertzanis *et al.*, 2009 ▸), each of which includes one or more steps to compare predicted crystal packings and remove duplicates (Day, 2011 ▸).

Each polymorph is defined by its space group, its lattice parameters and the atomic coordinates of its asymmetric unit. The asymmetric unit is a subset of the crystallographic unit cell that can be used to generate a complete unit cell using the symmetry operators of the space group. Throughout this work, comparisons are described in terms of clusters of *N* molecules, rather than more cumbersome terminology such as *N* asymmetric units. Constructing an optimal reproducible comparison of two crystal polymorphs is a challenge because simply superimposing a single molecule from each conformer does not quantify intermolecular orientations. For this reason, crystal packing coordinate root-mean-square deviations (RMSDs) generally consider a cluster of *N* molecules (denoted RMSD_*N*_), where *N* is often chosen to be ∼20. Coordinate RMSD_*N*_ increases with *N* because small discrepancies between the lattice parameters of two polymorphs are magnified as cluster size increases. The requirement to prioritize *N* molecules (or *N* times the number of molecules in the asymmetric unit when more than one molecule is present) from each polymorph and match them prior to calculation of the RMSD_*N*_ can lead to ambiguous results unless the shape of the superimposed clusters is reported via a simple metric such as radius of gyration (*R*_g_).

Multiple algorithms have been proposed to quantify crystal structure similarity. In addition to their own algorithm (named *CMPZ*), Hundt *et al.* (2006 ▸) presented a thorough history of early crystal comparison approaches. There are a plethora of crystal comparison algorithms currently available, using a variety of methods ranging from reductions in the dimensionality of input structures into more manageable representations based on intrinsic properties (*e.g.* periodic point sets, crystallographic information, X-ray powder diffraction *etc.*) to transformations of the crystallographic information into a many-dimensional configuration (or fingerprint) space (Sadeghi *et al.*, 2013 ▸; Valle & Oganov, 2010 ▸; Willighagen *et al.*, 2005 ▸; Gelder *et al.*, 2001 ▸; Karfunkel *et al.*, 1993 ▸; Verwer & Leusen, 1998 ▸; Mosca & Kurlin, 2020 ▸; Thomas *et al.*, 2021 ▸; Widdowson *et al.*, 2022 ▸; Edelsbrunner *et al.*, 2021 ▸; de la Flor *et al.*, 2016 ▸; Ferré *et al.*, 2015 ▸; Hicks *et al.*, 2021 ▸; De *et al.*, 2016 ▸; Gelato & Parthé, 1987 ▸; Dzyabchenko, 1994 ▸; Lonie & Zurek, 2012 ▸; Su *et al.*, 2017 ▸; Ong *et al.*, 2013 ▸). These methods can mitigate complexities that arise when dealing with a direct comparison of atomic positions (*e.g.* atom labeling, special positions, space group conversions *etc.*). However, comparisons produced via this approach can be difficult to visualize. Another genre of comparisons consists of overlapping packing shells (*i.e.* sub-clusters) of the desired crystals before calculating a metric that is usually based on distances and/or angles (Gelbrich & Hursthouse, 2005 ▸; Rohlíček & Skořepová, 2020 ▸; Rohlíček *et al.*, 2016 ▸; Chisholm & Motherwell, 2005 ▸).

A widely used algorithm that follows this final classification is *COMPACK* (Chisholm & Motherwell, 2005 ▸), which was proposed by the Cambridge Crystallographic Data Centre (CCDC, Cambridge, UK) (Groom *et al.*, 2016 ▸). *COMPACK* is maintained within the software program *Mercury* (Macrae *et al.*, 2020 ▸). *COMPACK* represents the molecular distribution of a specified number of molecules by recording interatomic distances and creates triangular subsets to generate a unique representation of a given crystal for comparison with other crystals. Two molecules within the clusters match when the difference between their distances is less than a specified distance tolerance (as a percentage) and the angles of their triangles differ by less than a specified angle tolerance (in degrees). This method quantifies crystal similarity regardless of the space group and lattice parameters. However, the implementation of the *COMPACK* algorithm is relatively slow and currently exhibits difficulties scaling up to large entities (*e.g.* proteins and nucleic acids).

In this study, we describe an algorithm for evaluating crystal packing similarity called *Progressive Alignment of Crystals* (*PAC*). This algorithm relies on a progressive series of coordinate superpositions to align *N* molecules. The algorithm performs similarly to *COMPACK* on small-molecule crystals but also scales up to biomolecular crystal comparisons. The implementation is faster than available alternatives using a single process and shows favorable parallel scaling to 64 processes. Finally, we introduce the use of metrics to quantify the shape of superimposed clusters (*e.g.* *R*_g_ and/or anisotropy) to avoid ambiguity when reporting results [*e.g.* for the CCDC blind assessment of crystal structure prediction (CSP)] and help to prioritize molecules during CSP workflows.

## 2. Materials

### 2.1. Software

The *PAC* algorithm is maintained within the *Force Field X* (*FFX*) software package that is freely available from GitHub (https://github.com/SchniedersLab/forcefieldx). Further documentation can be found on the Schnieders Laboratory website (https://ffx.biochem.uiowa.edu/). Like most programs in *FFX*, *PAC* is written in Java, invoked by a Groovy script, and requires Version 10 or later of the Java Development Kit. Further assistance for the installation process can be found at the GitHub link above.

The 2021 Cambridge Structural Database (CSD) software (Version 3.0.4) was utilized for the *COMPACK* comparisons. A default number of 20 molecules was chosen unless otherwise stated. All *COMPACK* comparisons were performed with a distance tolerance of 25% and an angle tolerance of 25°, unless higher values were necessary for the comparison to succeed (such cases will have the tolerances labeled). All single-process timing comparisons were performed using an Intel Core i7-9800X CPU (16 cores) at 3.80 GHz running x86_64.

### 2.2. Data for evaluating the *PAC* algorithm

**Figure 1:**
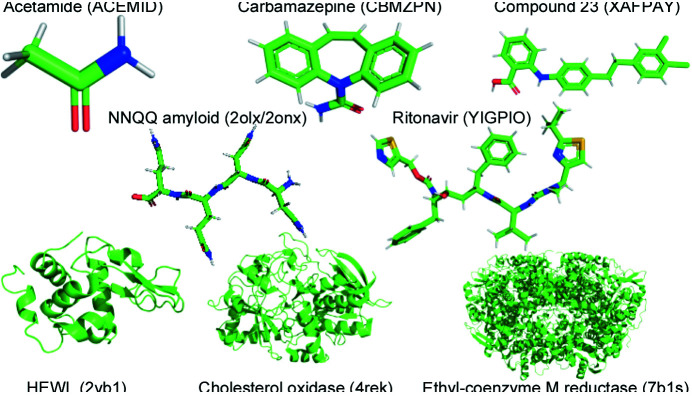
*PyMol* (Schrödinger, 2015 ▸) renderings of the molecules and proteins used to test the *PAC* algorithm. Structures with four alphanumeric characters are from the PDB and those with six letters are from the CSD.

We have designed the *PAC* algorithm to be applicable to a wide range of crystal structures. Therefore, the test crystals include molecules/proteins that scale in atom count (4–20 409 non-hydrogen atoms) and include both small-molecule and biological crystals. Each entity, depicted in Fig. 1 ▸, will be listed as follows: IUPAC name or abbreviation (database abbreviation; molecular formula; space groups).

The biological crystals in this study were obtained from the RCSB Protein Data Bank (PDB; http://www.rcsb.org/) (Berman *et al.*, 2000 ▸) and are used to demonstrate *PAC* on larger systems. Two polymorphs were selected for the NNQQ peptide (composed of two asparagine and two glutamine residues) of the yeast prion sup35 with 35 non-hydrogen atoms (2olx; C_18_H_30_N_8_O_9_; *P*2_1_2_1_2_1_) and (2onx; C_18_H_30_N_8_O_9_; *P*2_1_) (Sawaya *et al.*, 2007 ▸). The hen egg white lysozyme (HEWL) hydro­lase with 1001 non-hydrogen atoms (2vb1; *P*1) (Wang *et al.*, 2007 ▸) was chosen to represent small proteins and a cholesterol reductase from *Brevibacterium sterolicum* with 3834 non-hydrogen atoms (4rek; *P*2_1_) (Zarychta *et al.*, 2015 ▸) was selected as a midsize protein. The largest protein utilized in this study was ethyl-coenzyme M reductase from *Candidatus ethanoperedens thermophilum* with 20 409 non-hydrogen atoms (7b1s; *P*2_1_) (Hahn *et al.*, 2021 ▸). Both water and co-solutes were removed prior to applying *PAC*.

All the small molecules were accessed from the CSD (Groom *et al.*, 2016 ▸). The smallest mol­ecule included was acetamide with four non-hydrogen atoms (ACEMID; C_2_H_5_NO; *Pccn*, *H*3*c*). Carbamazepine (5*H*-dibenzo[*b*,*f*]azepine-5-carboxamide) with 18 non-hydrogen atoms (CBMZPN; C_15_H_12_N_2_O; *P*2_1_/*c*, *P*2_1_/*n*, 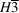 , 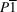 , *C*2/*c*, *Pbca*) serves as a classic example of crystal polymorphism (Reboul *et al.*, 1981 ▸; Arlin *et al.*, 2011 ▸; Lang *et al.*, 2002 ▸; Lowes *et al.*, 1987 ▸). The largest small molecule included in this study is ritonavir {[5*S-*(5R*,8R*,10R*,11R*)]-10-hydroxy-2-methyl-5-isopropyl-1-(2-isopropyl-4-thiazolyl)-3,6-dioxo-8,11-dibenzyl-2,4,7,12-tetraaza­tridecan-13-oic acid 5-thiazolyl methyl ester} with 50 non-hydrogen atoms (YIGPIO; C_37_H_48_N_6_O_5_S_2_; *P*2_1_, *P*2_1_2_1_2_1_). Additionally, the CCDC has hosted several blind crystal structure prediction (BCSP) competitions which allow participants to apply their algorithms to crystal structures determined via physical experiments (*e.g.* X-ray crystallography) which have yet to be released to the public. In the BCSP contest held in 2015, participants started from a two-dimensional chemical diagram and predicted one to two list(s) that contained up to 100 predicted crystal structures (Reilly *et al.*, 2016 ▸). Compound XXIII or 2-({4-[2-(3,4-di­chloro­phenyl)­ethyl]­phenyl}­amino)­benzoic acid with 26 non-hydrogen atoms (XAFPAY; C_21_H_17_Cl_2_N_1_O_2_; 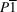 , *P*2_1_/*c*, *P*2_1_/*n*) (Samas, *et al.*, 2021 ▸) was selected to demonstrate how RMSD_*N*_ rank and *R*_g_ are affected for participant submissions based on the mol­ecular prioritization criterion for cluster inclusion (*i.e.* single linkage versus average linkage).

AMOEBA (Ponder *et al.*, 2010 ▸; Ren *et al.*, 2011 ▸) parameters were generated using the *PolType2* (Wu *et al.*, 2012 ▸; Walker *et al.*, 2022 ▸) automatic parameterization program on SDF files obtained from PubChem (Kim *et al.*, 2021 ▸). Local optimization of coordinates and lattice parameters of each experimental structure to an energetic convergence criterion of 0.1 kcal mol^−1^ Å^−1^ (1 kcal mol^−1^ = 4.184 kJ mol^−1^) was performed according to AMOEBA using *Force Field X*. The AMOEBA minimization produced crystal polymorphs that were compared with experimental structures using both *COMPACK* and *PAC*.

## 3. The *PAC* algorithm

**Figure 2:**
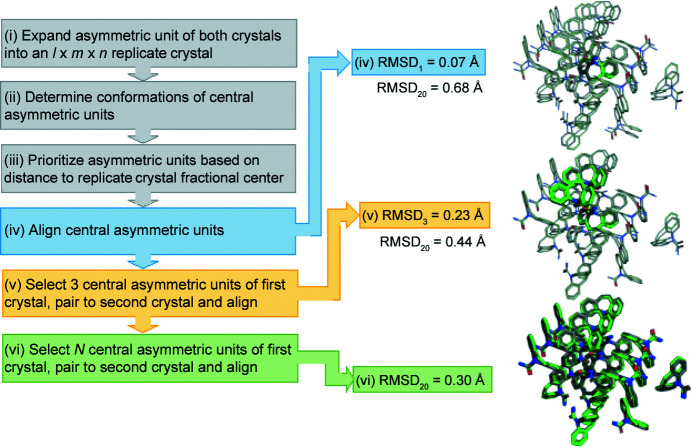
A general overview of the *PAC* algorithm, which consists of a progressive series of alignments to optimize RMSD_*N*_ between superimposed clusters with *N* molecules. The six basic steps for the algorithm are listed in the flow chart on the left, with crystal alignments emphasized as superimposed images on the right. This example comparison was performed using single linkage to prioritize the addition of molecules into the clusters. The RMSD between similar crystals improves as the alignment progresses.

The six main steps to compare two crystals according to the *PAC* algorithm follow the flow chart and images in Fig. 2 ▸ (images and values obtained from single linkage comparison). All alignments in this algorithm are performed via quaternion superposition (Horn, 1987 ▸; Kearsley, 1989 ▸). Inputs to *PAC* include the atomic coordinates of atoms in the asymmetric unit, the space group and the lattice parameters for two crystals. Although *PAC* can handle multiple molecules/proteins in the asymmetric unit, for simplicity the algorithm will be described assuming that the asymmetric unit contains a single molecule. A subset of atoms can be selected for the comparison (*e.g.* non-hydrogen atoms, α-carbons *etc.*), which will be more thoroughly described in the *Discussion* [Sec sec5] section below. Mass weighting can be utilized, but comparisons in this work were performed utilizing geometric centers. By default, *PAC* does not use mass weighting, to avoid overprioritizing third period or higher elements (*e.g.* phosphorus, chlorine *etc.*) relative to second period elements. Hydrogen atoms are not included by default as their experimental coordinates are often more uncertain than those for heavier atoms.

(i) The molecular coordinates from each structure are expanded through the crystallographic information provided until each crystal occupies a scalar (default of six) times the expected volume of the final cluster. The expected volume for an RMSD_*N*_ is calculated by dividing the volume of the unit cell by the number of molecules it contains and multiplying by *N*.

(ii) The unique molecules are paired between crystals on the basis of a molecular RMSD (*i.e.* RMSD_1_). The number of unique molecules in each crystal is determined according to the space group and the number of molecules in the asymmetric unit (*Z*′). Crystals in a Sohncke space group are non-enantiogenic (*i.e.* do not create a non-superimposable copy of the entity) and will have the same number of conformations as *Z*′. However, enantiogenic space groups create 2 × *Z*′ conformations. Therefore, *PAC* loops through the molecules in each crystal (prioritizing molecules closest to the center) and identifies the unique molecular conformations in each crystal.

(iii) Molecules are then ranked by the distance of their geometric center from the center of all atoms in the expanded crystal.

(iv) Both crystals are translated so the geometric centers of their center-most molecules are at the origin. The central molecule of the second crystal is rotated to achieve optimal superposition on that from the first crystal. For the example in Fig. 2 ▸, the central molecule has an RMSD_1_ of 0.068 Å, whereas RMSD_20_ at this stage is 0.684 Å.

(v) The second and third closest molecules from the first crystal (using a specified linkage criterion discussed below) are matched via geometric distance to molecules within the second crystal. The alignment of the two crystals is based on the three molecules that have been matched between the crystals. RMSD_3_ in Fig. 2 ▸ for this alignment is 0.227 Å, while RMSD_20_ has been reduced to 0.444 Å.

(vi) Finally, *N* molecules closest to the central molecule of the first crystal are matched with those from the second crystal and a final coordinate alignment is performed. Coordinates for the selected atoms produced from this final alignment are utilized to compute RMSD_*N*_. Using this procedure, the example in Fig. 2 ▸ has an RMSD_20_ of 0.302 Å.

The selected molecules for the cluster of the first crystal are known prior to consideration of the second crystal because selection is based only on the linkage method (linkage description given below). However, the selected molecules for the cluster of the second crystal depend on the distances between the molecules of the two crystals, which change during the alignment performed in steps (iv), (v) and (vi) above. If the crystals are sufficiently similar (*e.g.* the example used in Fig. 2 ▸), then the selected *N* molecules for the cluster of the second crystal remain the same and RMSD_*N*_ progressively decreases. Steps (iv)–(vi) are repeated for each pair of unique molecules between the two crystals. The final RMSD_*N*_ between the compared crystals is the minimum value produced from the repeated comparisons.

The *PAC* algorithm supports three linkage criteria, which follow those widely used for hierarchical clustering, to select molecules for cluster inclusion:

(*a*) single (shortest atomic distance between two mol­ecules)

(*b*) average (shortest distance between the average atomic positions of two molecules)

(*c*) complete (shortest atomic distance for the most widely separated atoms between two molecules)

**Figure 3:**
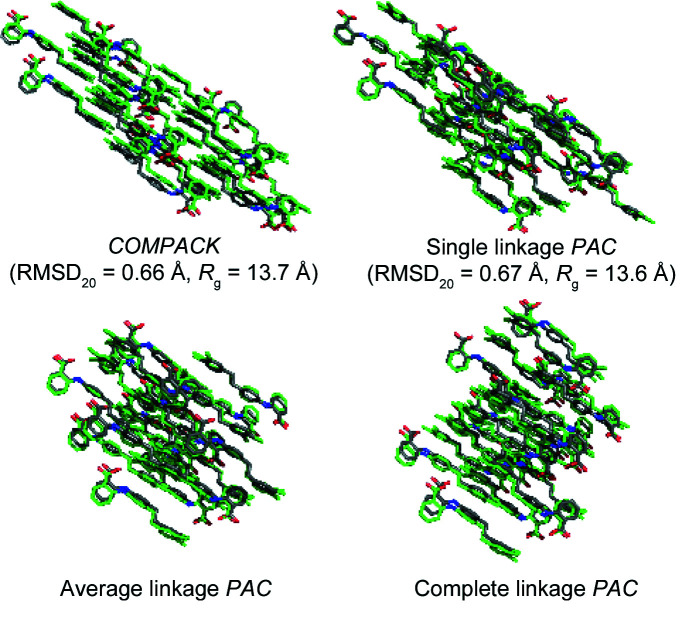
Different linkage methods affect the molecular cluster shape, RMSD_20_ and radius of gyration (*R*_g_).

Depending on the selected linkage criterion, the final cluster shape and RMSD_*N*_ usually differ, as shown in Fig. 3 ▸.

Structure metrics have previously been used to characterize proteins to assess characteristics of their 3D structures (Šolc, 1971 ▸; Blavatska & Janke, 2010 ▸). The gyration tensor quantifies the deviation of atoms from the geometric center (GC) of all atoms within the cluster,

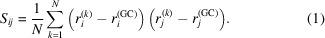




The elements of the gyration tensor [*S_ij_* from equation (1)] are defined as the sum of the coordinate distances to the geometric center for each of *N* atoms where *i* and *j* denote the *x*, *y* or *z* coordinate.

The principal moments of the gyration tensor (with eigenvalues λ_min_, λ_med_ and λ_max_) equate to the squared characteristic semi-axis lengths that describe the ellipsoid containing the cluster of atoms. The sum of the principal moments results in the squared *R*_g_,

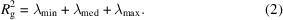

Reporting *R*_g_ along with RMSD_*N*_ quantifies whether or not the packing comparison has achieved a cluster geometry that equally weights each crystal axis. For the structures compared in this study, single linkage performs most similarly to *COMPACK*, but average linkage generally provides a preferable compromise between low RMSD_*N*_ and low *R*_g_. Other descriptive metrics such as moments of inertia, asphericity, acyl­indricity and anisotropy are also reported by the *PAC* algorithm, but *R*_g_ is generally sufficient to assess the impact of linkage choice. All data generated via complete linkage are given in the supporting information.

## 4. Results

### 4.1. Accuracy

**Figure 4:**
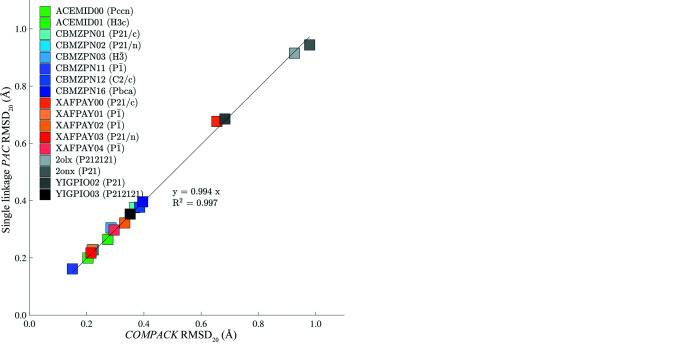
Output metrics for *COMPACK* are plotted on the *x* axis and *PAC* results are plotted on the *y* axis. (*a*) *PAC* with single linkage produces similar RMSD_20_ values to *COMPACK*, as demonstrated by the regression slope of 0.994. (*b*) The RMSD_20_ values for *PAC* with average linkage tend to be slightly larger than those for both *COMPACK* and *PAC* with single linkage.

Each of the experimentally determined structures listed in *Materials* [Sec sec2] was compared with minimized coordinates and lattice parameters (minimization via the AMOEBA force field) utilizing *COMPACK*, *PAC* with single linkage and *PAC* with average linkage. The comparisons were performed at a comparison shell size of 20 molecules and did not include hydrogen atoms. The RMSDs between the experimental crystals and AMOEBA lattice-minimized crystals are plotted in Fig. 4 ▸.

**Figure 5:**
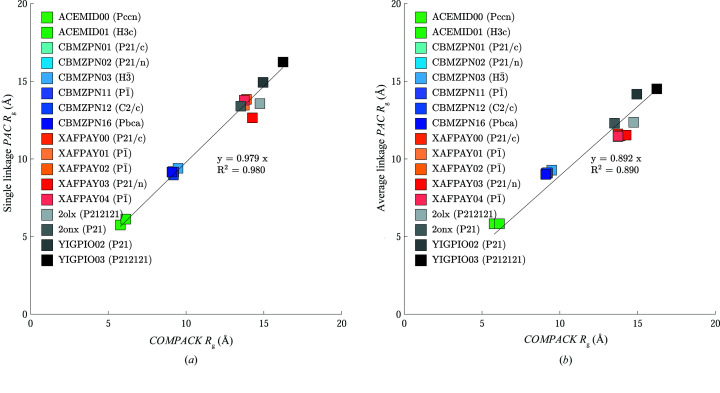
Crystal packing comparison algorithms use a range of criteria to prioritize molecules for inclusion in superimposed clusters, which affects both RMSD_20_ and cluster shape as quantified by radius of gyration *R*_g_. (*a*) *R*_g_ values from *COMPACK* are similar to those from *PAC* with single linkage, based on clusters selected for RMSD_20_. (*b*) Radius of gyration values from *PAC* with average linkage are significantly smaller.

The average *R*_g_ was calculated for each pair of clusters generated in the comparisons that produced Fig. 4 ▸. The *R*_g_ values for these comparisons are plotted in Fig. 5 ▸.

We obtained the crystal submissions from the 2015 BCSP exercise and reproduced the *COMPACK* comparisons (20 molecule shells, distance tolerance of 25% and angle tolerance of 25°). The crystal structures that successfully produced RMSD_20_ values for *COMPACK* relative to the experimentally determined polymorphs for XAFPAY were also compared with *PAC*. The results of the 2015 BCSP competition focused on the ability of contestants to rank their own submissions (*i.e.* the team that ranked a submission with an RMSD < 0.8 Å higher than another group was considered to have a better prediction, regardless of the experimental RMSD). The ability of the contestants to predict experimental structures accurately (*i.e.* to produce crystals that obtain a low RMSD) is also important. Table 1 ▸ contains the RMSD_20_ values for the experimental structure XAFPAY01 (polymorph *B*) from *COMPACK* and *PAC* using average linkage (the corresponding data for single and complete linkages can be found in the supporting information, Table S2). Two such crystal comparisons that were originally included in the supporting information of the BCSP paper were not reproducible with our version of *COMPACK* at the reported tolerances. Therefore, we used the values reported previously and replaced the *R*_g_ for the clusters with a dash. The structures are ordered on the basis of the computed *COMPACK* RMSD_20_ and their corresponding ranks are presented for *PAC* using average linkage. Additionally, the average *R*_g_ between the compared molecular clusters is reported for each comparison. These *PAC* comparisons were completed on the Fugaku supercomputer at the Riken Center for Computational Science in Kobe, Japan.

### 4.2. Performance

**Figure 6:**
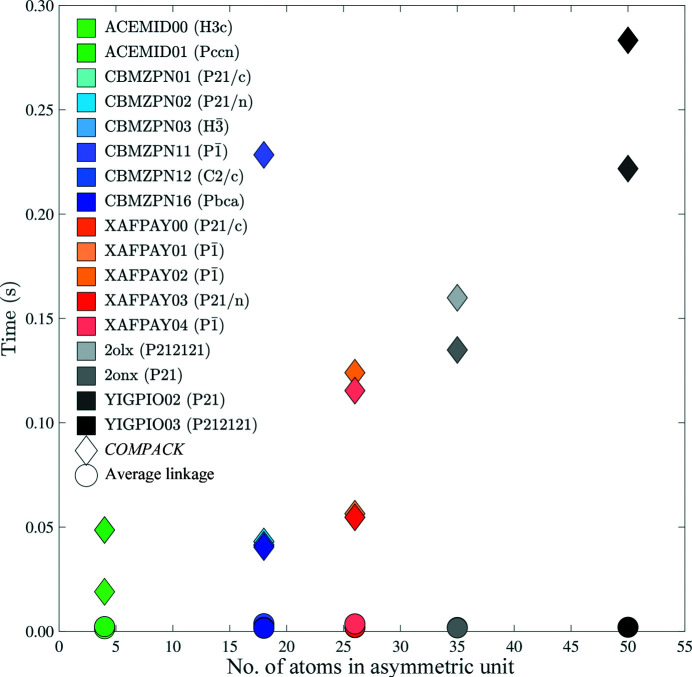
Packing comparison computational cost increases with number of atoms. *COMPACK* and *PAC* timings are represented by diamonds and circles, respectively. Each entity is color coded according to the legend. The time presented is the fastest out of 100 RMSD_20_ trials.

*COMPACK* and *PAC* were used to perform all versus all comparisons between 100 crystal structures obtained from a molecular dynamics simulation on the experimental crystal structure using the AMOEBA force field. Relative to *COMPACK*, all *PAC* linkage methods display similar comparison times, and therefore average linkage will be presented for all figures in the main text. Timing figures utilizing single and complete linkage are included in Figs. S3–S5. The times presented in Fig. 6 ▸ are the fastest elapsed CPU times for a single 20-molecule comparison when comparing each of the 100 structures generated from the simulation with themselves (total 10 000 comparisons).

**Figure 7:**
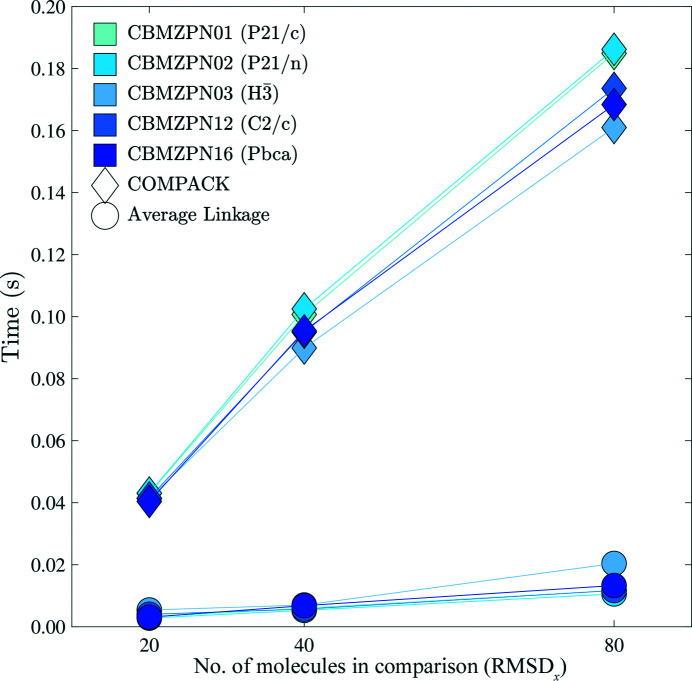
Packing comparison computational cost increases with the number of molecules *N* included in the cluster. *COMPACK* and *PAC* are represented by diamonds and circles, respectively. The time presented is the fastest out of 100 identical trials.

The 100 molecular dynamics snapshots for each carbamazepine crystal underwent all versus all RMSD_*N*_ packing comparisons for increasing values of *N* = {20, 40, 80}, with the results shown in Fig. 7 ▸ (other molecules display similar trends). CBMZPN11 ( 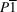 ) was left out of the graph as the *COMPACK* timings extend above 0.2 s and would lower its resolution. All *PAC* comparisons were at least eight times faster than the corresponding *COMPACK* timings.

**Figure 8:**
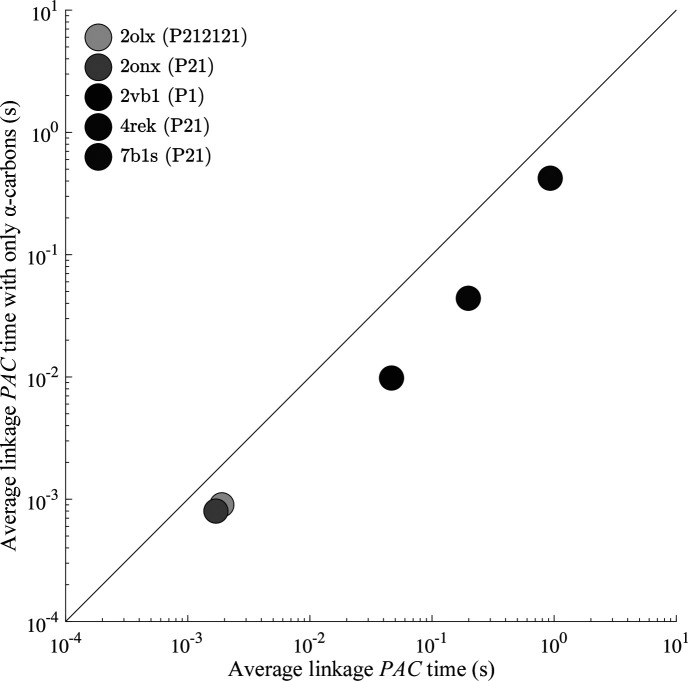
Comparisons using a specified subset of atoms can significantly reduce the calculation time. The durations shown are the fastest RMSD_20_ comparison out of 100 trials between two protein crystals. The abscissa represents RMSD_20_ values for the default *PAC* algorithm and the ordinate depicts the RMSD_20_ for a comparison limited to α-carbons. Log scales are utilized to allow all protein comparisons to be displayed on the same graph.

As seen in Figs. 6 ▸ and 7 ▸, an increase in the number of atoms within a cluster increases the computational time necessary to perform a packing comparison. Therefore, it is useful to restrict the number of atoms being compared when possible. In addition to limiting comparisons to non-hydrogen atoms, *PAC* can operate on protein α-carbon atoms or a custom subset. The use of α-carbon atoms significantly decreases the duration of each comparison, as shown in Fig. 8 ▸.

**Figure 9:**
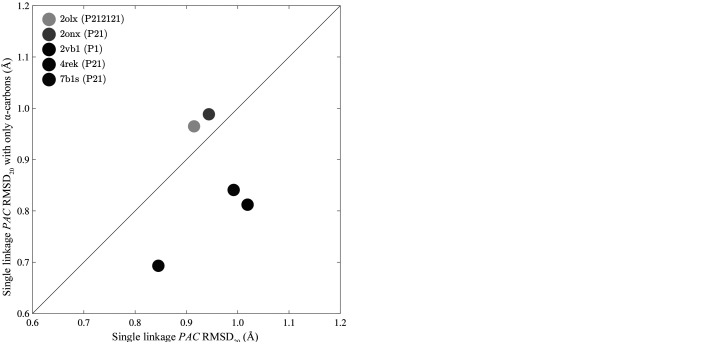
Restricting protein comparisons to consider only α-carbons results in a modest change in the RMSD_20_ values for the *PAC* algorithm. The abscissa shows RMSD_20_ values when using all heavy atoms for the comparison, while the ordinate is restricted to α-carbons. (*a*) Results with single linkage and (*b*) data using average linkage.

The RMSD values of the protein crystal comparisons change moderately through exclusion of side chains, as shown in Fig. 9 ▸.

**Figure 10:**
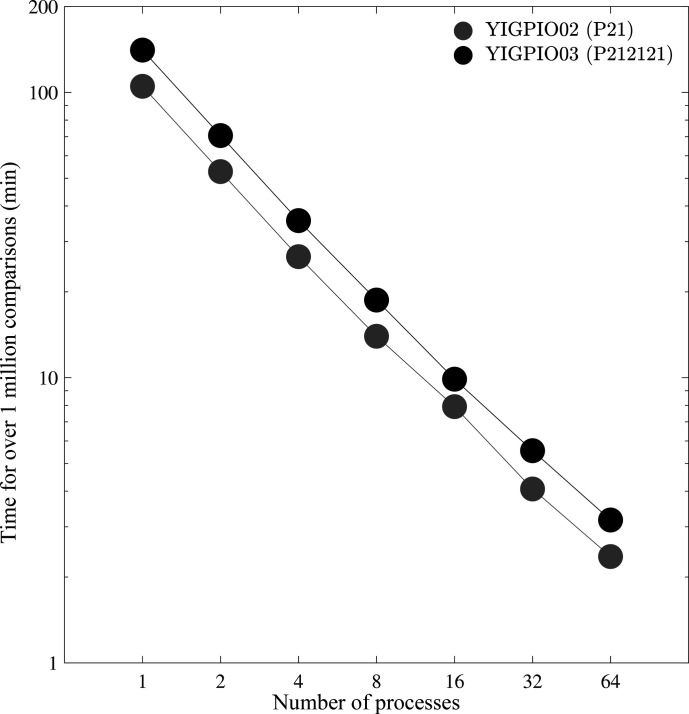
Ritonavir packing comparison performance is shown for the *PAC* algorithm when utilizing 1 to 64 processes. The ordinate shows the wall clock time necessary for *PAC* to perform over one million (1 048 576) comparisons, with the number of processes given on the abscissa.

The *PAC* algorithm can divide comparisons between multiple processes. The comparisons of the 100 molecular dynamics snapshots (RMSD_20_ excluding hydrogen) were scaled up to an all versus all comparison of 1024 structures (for a total of 1 048 576 comparisons). The parallel comparisons were performed utilizing the Argon HPC cluster maintained at the University of Iowa, with nodes containing two Intel Xeon E5-2680 v4 CPUs at 2.40 GHz. Each parallel comparison (regardless of the number of processes) was allocated three 512 GB memory nodes, which consisted of 56 hyperthreaded cores (28 physical cores). Two hyperthreaded cores were assigned to each process, which limited each Argon node to a maximum of 28 processes. Algorithm logging was reduced and comparison results were written to a text file to promote maximum efficiency. The same *PAC* comparisons were performed while doubling the number of processes, as shown in Fig. 10 ▸. *PAC* presents moderately decreasing efficiency gains as more nodes are utilized, ranging from 1.96× speed-up with two nodes to 33.9× speed-up with 64 nodes (∼53% efficiency, resulting in more than 3000 comparisons per second at 64 nodes).

## 5. Discussion

Crystal packing comparison methods compute the coordinate RMSD_*N*_ for a cluster of *N* molecules, but the shape of the compared clusters is typically not reported. While the lowest possible RMSD_*N*_ may result from elongated clusters that prioritize accurate packing along a single dimension, uniform prioritization of packing in all three dimensions serves to minimize the radius of gyration. Just as the global distance test (GDT) is of central importance in the critical assessment of structure prediction (Moult *et al.*, 1995 ▸), so RMSD_*N*_ serves as the gold standard for comparing entries in the CCDC CSP blind tests with experiment. By reporting *R*_g_ along with RMSD_*N*_, the shape of the compared clusters (*i.e.* elongated versus spherical) can be appreciated and ambiguity reduced. Generally, single linkage yields lower RMSD_*N*_ at the cost of higher *R*_g_ and more closely replicates *COMPACK* [Figs. 4 ▸(*a*) and 5 ▸(*a*)]. According to the data reported here, average linkage results in clusters that more equally prioritize all three dimensions and thereby lowers *R*_g_ with only modestly higher RMSD_*N*_ values [Figs. 4 ▸(*b*) and 5 ▸(*b*)].

As seen in Table S2, the order of crystals based on RMSD changes minimally between *COMPACK* and *PAC* with single linkage. However, in Table 1 ▸, average linkage has several structures whose rank increases significantly (highlighted in bold). Each of the highlighted predictions had their rank increase by at least 15 places when using average linkage, which shows that their crystal packing is more closely related to experiment when spherical clusters are prioritized. Furthermore, a series of crystals featuring molecules with an increasing number of methyl groups between two acetamides were compared to observe the effect of molecule length on *R*_g_ (values in Table S6). The *R*_g_ values for selected clusters increase with molecule length regardless of the comparison method selected, although average linkage shows less variation than *COMPACK* or single linkage. Size alone may not fully describe the differences in the values of *R*_g_. For example, the protein crystals utilized in this study have very similar *R*_g_. However, the molecules in the diacetamide crystals (and XAFPAY polymorphs) are relatively linear, which might promote preferential selection in *COMPACK* and single linkage. The incorporation of *R*_g_ improves the robustness of *PAC* by encouraging a selection of molecules that do not favor a specific orientation. When the unit-cell volumes differ dramatically between two crystals, it is possible that *PAC* (and *COMPACK*) can inappropriately quantify the crystal similarity with a low RMSD if large sections of the two crystals are similar (Table S2). Increasing the number of molecules included in the comparison can improve the fidelity of *PAC* with a modest loss in efficiency. Multiplying the default number of molecules by a factor of volume change worked well for the provided test systems (*e.g.* if one unit cell is roughly four times greater than the other, then a comparison cluster of 80 molecules could be used).

The efficiency increase of the *PAC* algorithm has implications for crystal structure prediction, where many candidate packings are generated and must be compared. Relative to *COMPACK*, the computational cost of *PAC* comparisons scales more favorably as the number of atoms increases, which allows it to scale up to larger crystals (*e.g.* proteins, nucleic acids *etc.*). *PAC* also maintains efficiency for packing comparisons as the number of molecules *N* increases (Figs. 6 ▸ and 7 ▸). Finally, *PAC* leverages the non-enantiomorphic nature of Sohncke groups featured in most biological crystals for additional efficiency. Inclusion of all non-hydrogen atoms in the packing comparison is recommended when efficiency is not a limiting factor, but the ability to select a subset of atoms provides performance improvements (Figs. 8 ▸ and 9 ▸). For example, the exclusion of side-chain atoms tends to slightly reduce the RMSD_*N*_ for large proteins, as the algorithm focuses exclusively on the alignment of the amino acid backbone conformation. The *PAC* algorithm is parallelized over processes using MPI to accelerate the performance of large batches of comparisons. Comparison times can be significantly reduced using parallel processors (Fig. 10 ▸). Furthermore, average linkage has improved efficiency over the other *PAC* linkage methods (single and complete) as all the atoms per constituent are condensed into a single point, which vastly reduces the number of distances that need to be evaluated.

## 6. Conclusions

We have proposed the *PAC* algorithm for evaluating the similarity of two crystal structures. The results demonstrate that *PAC* is an accurate and efficient method to evaluate the similarity of two crystal structures. *PAC* employs a progressive series of coordinate alignments to optimize RMSD_*N*_. The RMSD_*N*_ values obtained by *PAC* agree with those obtained from the widely used program *COMPACK* when using single linkage to prioritize molecules for inclusion in the superimposed clusters. *PAC* performed an average of 15 times faster than *COMPACK* when computing multiple comparisons for the carbamazepine polymorphs.

We suggest that the utilization of cluster shape metrics such as radius of gyration helps to avoid the ambiguity inherent in reporting RMSD_*N*_ alone.

*PAC* has many potential applications, including identification and removal of duplicate crystal structure candidates during CSP and the comparison of optimized structures with experimental data.

## Supplementary Material

Additional figures and tables. DOI: 10.1107/S1600576722009670/tu5028sup1.pdf

## Figures and Tables

**Table 1:** RMSD_20_ values for packing comparisons between experiment (XAFPAY01) and submissions to the CCDC’s 2015 BCSP assessment, showing how they depend on the algorithm used The rankings for many entries using *PAC* with average linkage are similar to those from *COMPACK*, but in some cases the rankings deviate significantly (highlighted in bold).

	*COMPACK*			*PAC*, average linkage		
Submission: BCSP team (rank R, list L)	Rank	RMSD_20_ (Å)	*R*_g_ (Å)	Rank	RMSD_20_ (Å)	*R*_g_ (Å)
Neuman, Kendrick, Leusen (R26, L2)	1	0.218	13.37	1	0.323	11.37
Neuman, Kendrick, Leusen (R04, L2)	2	0.229	13.37	2	0.328	11.36
Neuman, Kendrick, Leusen (R02, L1)	2	0.229	13.41	2	0.328	11.36
Price *et al.* (R05, L1)	4	0.286	15.92	4	0.359	11.38
Tkatchenko *et al.* (Price) (R05, L2)	5	0.294	14.20	5	0.435	11.34
Tkatchenko *et al.* (Price) (R02, L1)	5	0.294	14.22	5	0.435	11.34
Brandenburg & Grimme (Price) (R04, L2)	7	0.330	14.29	10	0.498	11.31
Brandenburg & Grimme (Price) (R08, L2)	8	0.334	14.23	9	0.469	11.32
Price *et al.* (R02, L2)	9	0.339	15.70	8	0.444	11.38
Price *et al.* (R01, L1)	10	0.340	15.74	7	0.442	11.38
Brandenburg & Grimme (Price) (R02, L2)	11	0.349	14.34	12	0.529	11.31
Brandenburg & Grimme (Price) (R03, L2)	12	0.369	14.32	16	0.550	11.31
Brandenburg & Grimme (Price) (R01, L2)	13	0.391	14.36	18	0.573	11.35
Brandenburg & Grimme (Price) (R26, L1)	14	0.392	15.54	17	0.554	11.30
Brandenburg & Grimme (Price) (R31, L1)	15	0.394	15.36	27	0.625	11.35
Brandenburg & Grimme (Price) (R06, L2)	16	0.396	14.25	19	0.586	11.32
Brandenburg & Grimme (Price) (R37, L1)	17	0.403	14.91	34	0.648	11.35
Brandenburg & Grimme (Price) (R38, L1)	18	0.405	14.85	20	0.589	11.30
Brandenburg & Grimme (Price) (R45, L1)	19	0.409	14.64	35	0.657	11.35
Brandenburg & Grimme (Price) (R07, L2)	20	0.412	14.23	24	0.613	11.35
Brandenburg & Grimme (Price) (R39, L1)	21	0.412	14.79	23	0.608	11.30
Brandenburg & Grimme (Price) (R05, L2)	22	0.414	14.27	22	0.601	11.35
Brandenburg & Grimme (Price) (R57, L1)	23	0.416	14.44	31	0.632	11.31
Brandenburg & Grimme (Price) (R34, L1)	24	0.418	15.09	25	0.618	11.30
Brandenburg & Grimme (Price) (R36, L1)	25	0.420	14.99	37	0.675	11.35
Brandenburg & Grimme (Price) (R32, L1)	26	0.421	15.20	26	0.624	11.30
Brandenburg & Grimme (Price) (R46, L1)	27	0.424	14.60	38	0.683	11.35
Brandenburg & Grimme (Price) (R61, L1)	28	0.425	14.39	30	0.628	11.30
Brandenburg & Grimme (Price) (R47, L1)	29	0.426	14.56	33	0.644	11.31
Brandenburg & Grimme (Price) (R59, L1)	30	0.427	14.42	28	0.628	11.30
Brandenburg & Grimme (Price) (R56, L1)	31	0.428	14.47	29	0.628	11.30
van Eijck (R20, L1)	32	0.430	14.23	**13**	0.533	11.47
Brandenburg & Grimme (Price) (R52, L1)	33	0.434	14.51	32	0.639	11.30
Elking & Fusti-Molnar (R78, L1)	34	0.434	14.23	**14**	0.536	11.43
Brandenburg & Grimme (Price) (R42, L1)	35	0.437	14.72	39	0.701	11.35
Brandenburg & Grimme (Price) (R44, L1)	36	0.448	14.68	36	0.658	11.30
Pantelides, Adjiman *et al.* (R21, L1)	37	0.455	14.08	**15**	0.544	11.40
Obata & Goto (R13, L1)	38	0.495	14.22	**21**	0.595	11.54
Brandenburg & Grimme (Price) (R11, L1)	39	0.524	–	**11**	0.515	11.33
Day *et al.* (R75, L2)	40	0.601	14.19	40	0.741	11.48
Pantelides, Adjiman *et al.* (R13, L1)	41	0.604	13.37	41	0.793	11.45
Mohamed (R88, L1)	42	0.827	–	42	0.843	11.55
						
Average values	–	0.408	14.52	–	0.573	11.36
