# Supplementary material for: Progressive alignment of crystals: reproducible and efficient assessment of crystal structure similarity
Source: J Appl Crystallogr. 2022 Nov 21;55(Pt 6):1528–37. doi: 10.1107/S1600576722009670 (PMC9721330; doi:10.1107/S1600576722009670)
Supplement: Supplementary file 1 [file j-55-01528-sup1.pdf]

JOURNAL OF
APPLIED
CRYSTALLOGRAPHY

Volume 55 (2022)

Supporting information for article:

Progressive alignment of crystals: reproducible and efficient assessment of crystal structure similarity

Aaron J. Nessler, Okimasa Okada, Mitchell J. Hermon, Hiroomi Nagata and Michael Schnieders

Supplementary Information

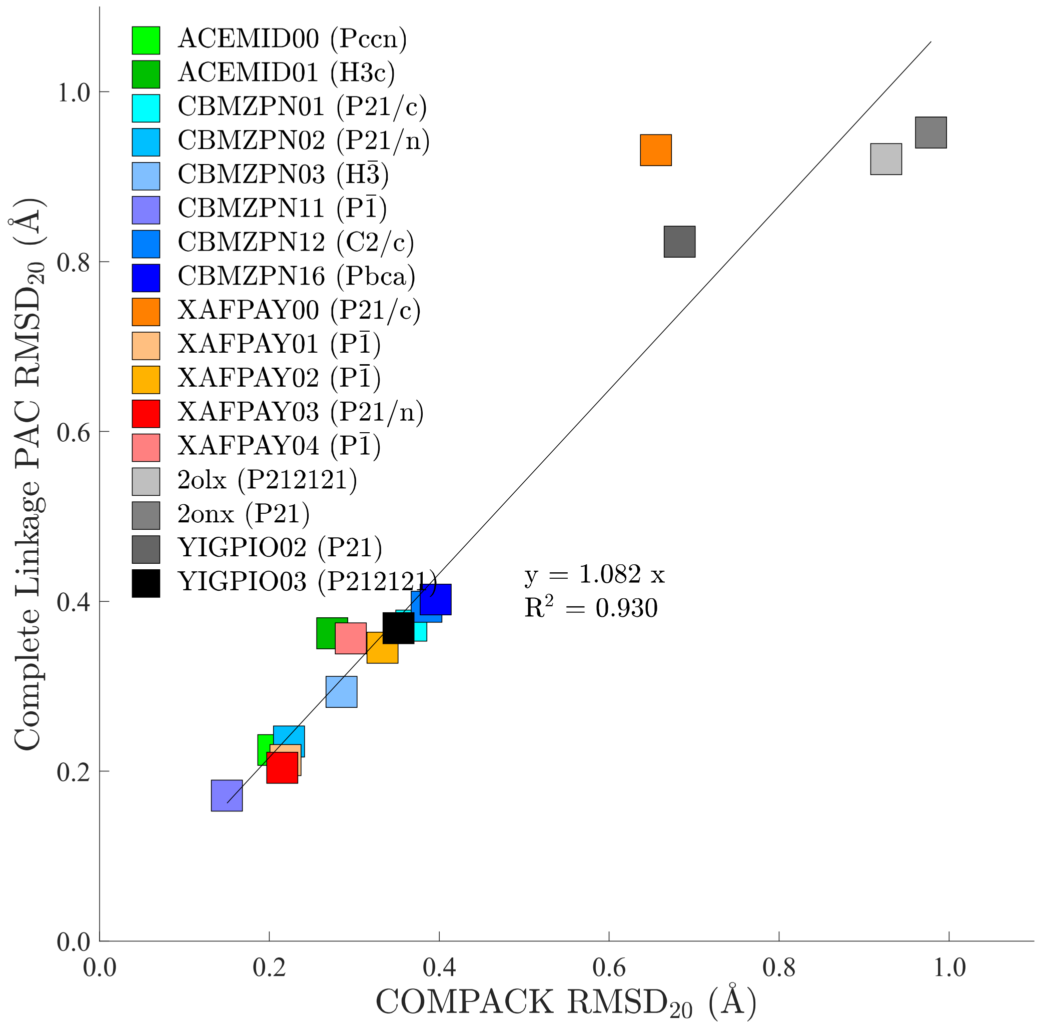

Figure S1. Comparing the use of complete linkage to select asymmetric units with COMPACK produces the RMSD plot above.

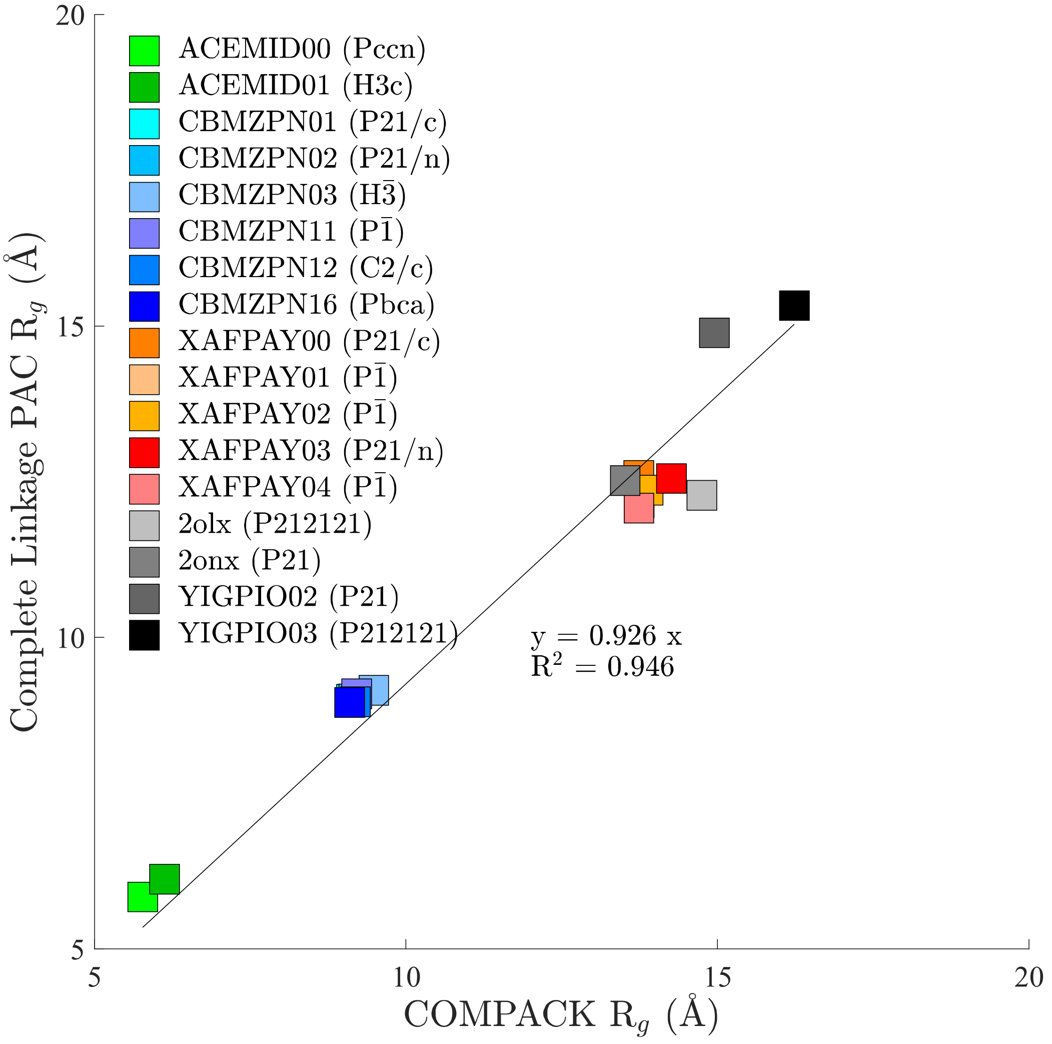

Figure S2. The use of complete linkage to select asymmetric units and comparing the *Rg* values produces the plot above.

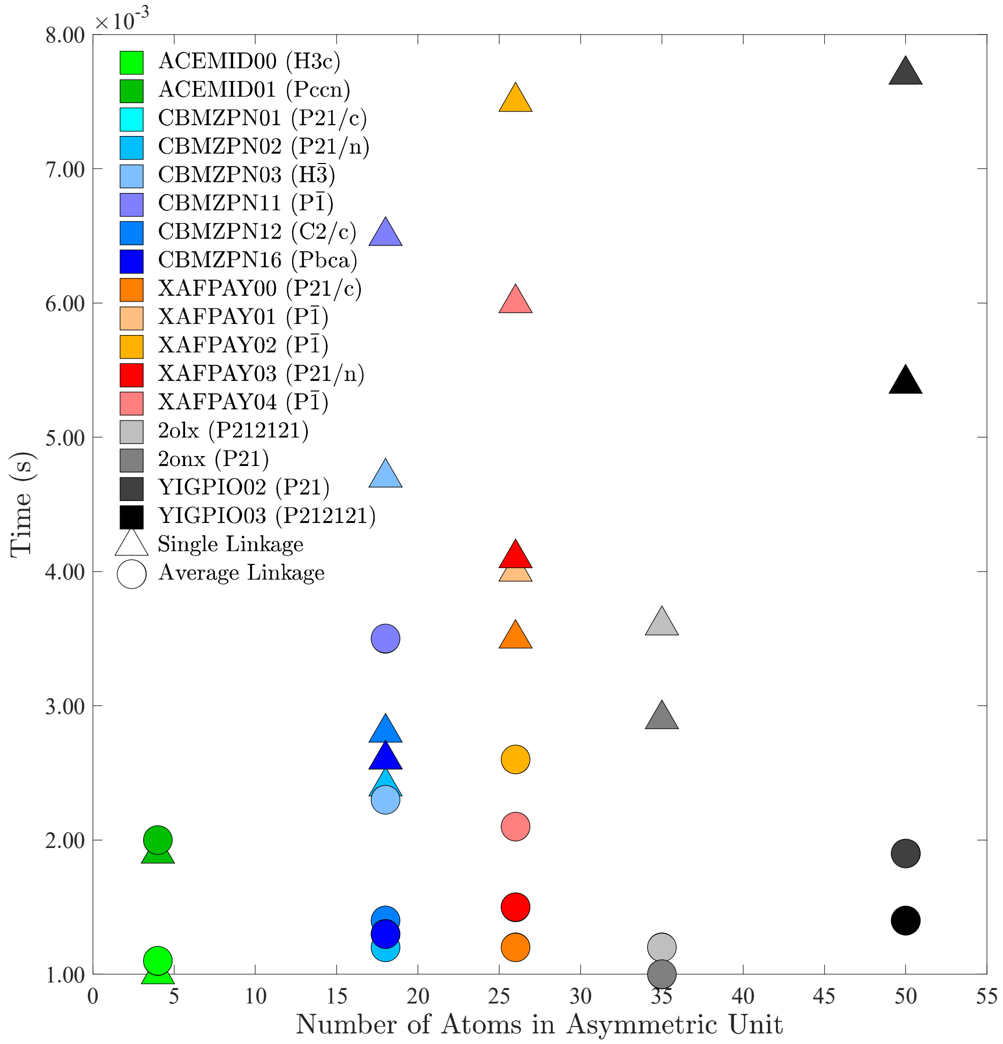

Figure S3. Comparison times for each of the linkages are relatively similar when compared to COMPACK. Although single and complete linkage have very similar comparison times, average linkage shows reduced comparison times when applied to larger systems.

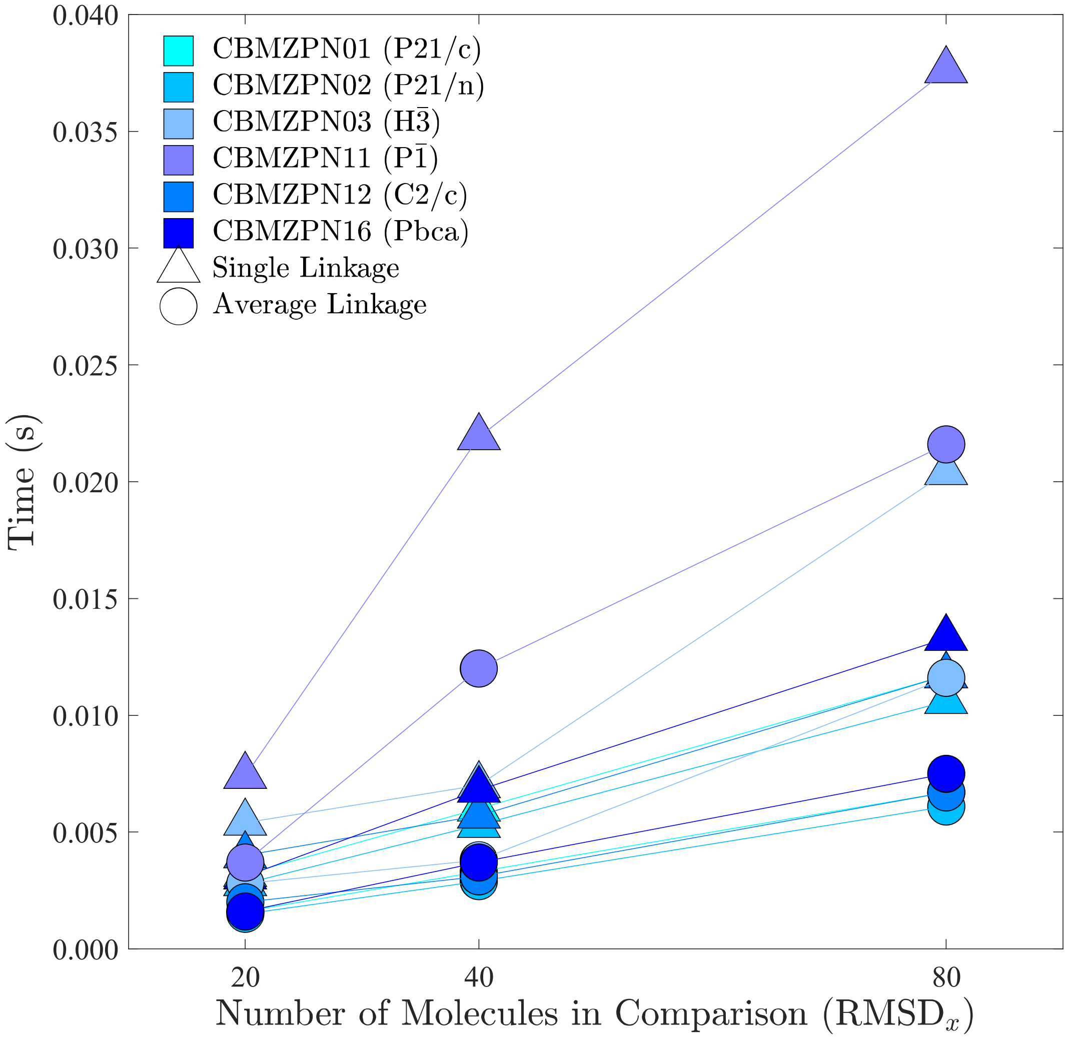

Figure S4. Increasing the number of molecules included in the comparison (*RMSD*$_x$) results in slight differences between single and average linkages. Timings for single and complete linkages are essentially equivalent.

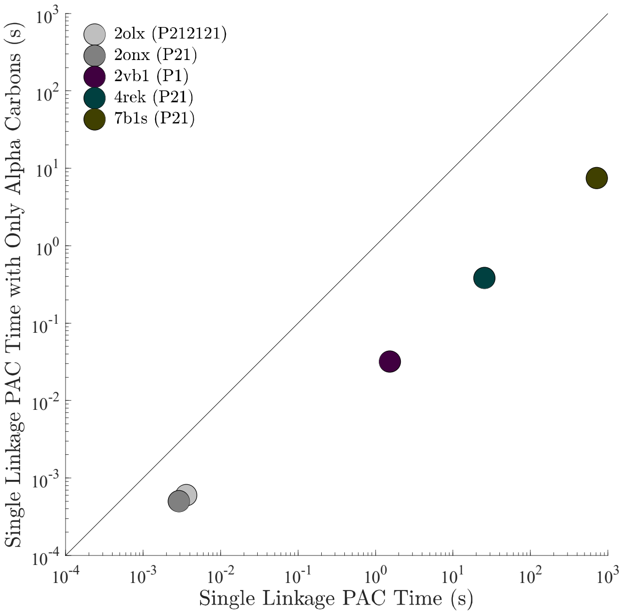

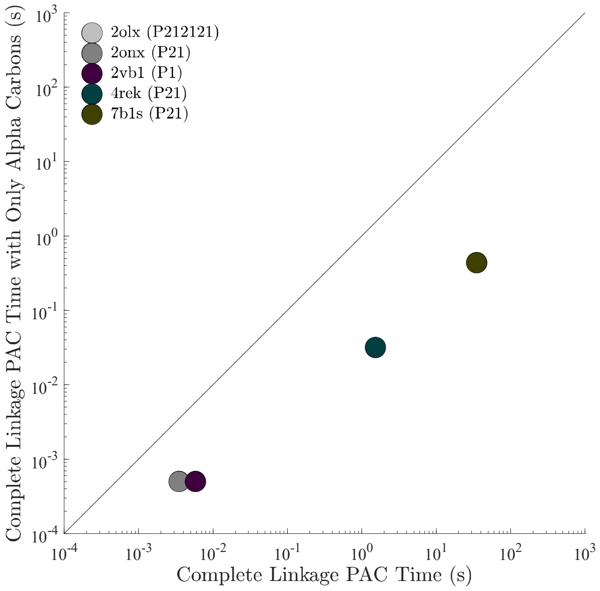

Figure S5. Comparisons gain a similar increase to efficiency when limiting protein comparisons to alpha carbons regardless of linkage used. The top panel uses single linkage whereas the bottom panel uses complete linkage.

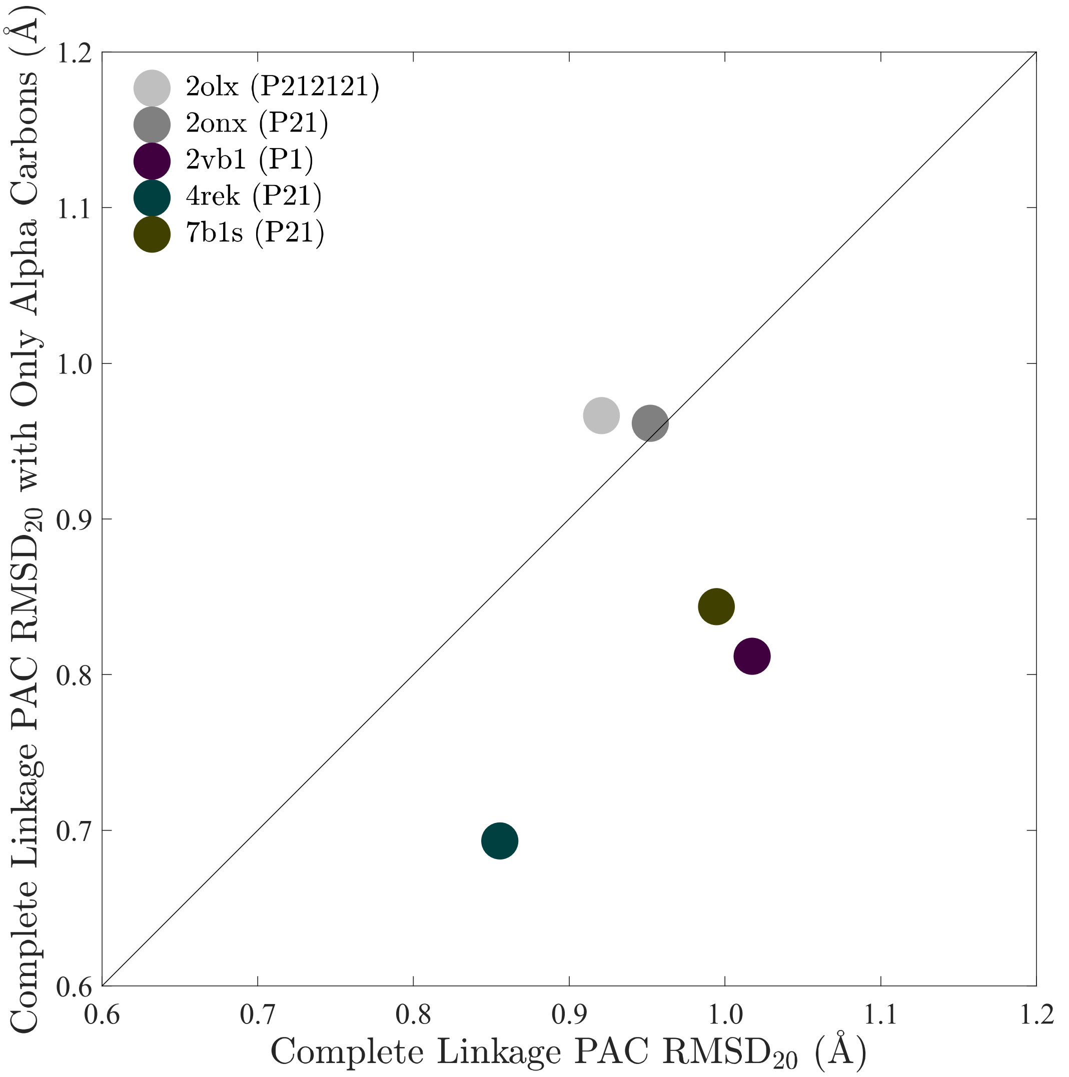

Figure S6. This figure demonstrates the accuracy change for utilizing only alpha carbons with complete linkage.

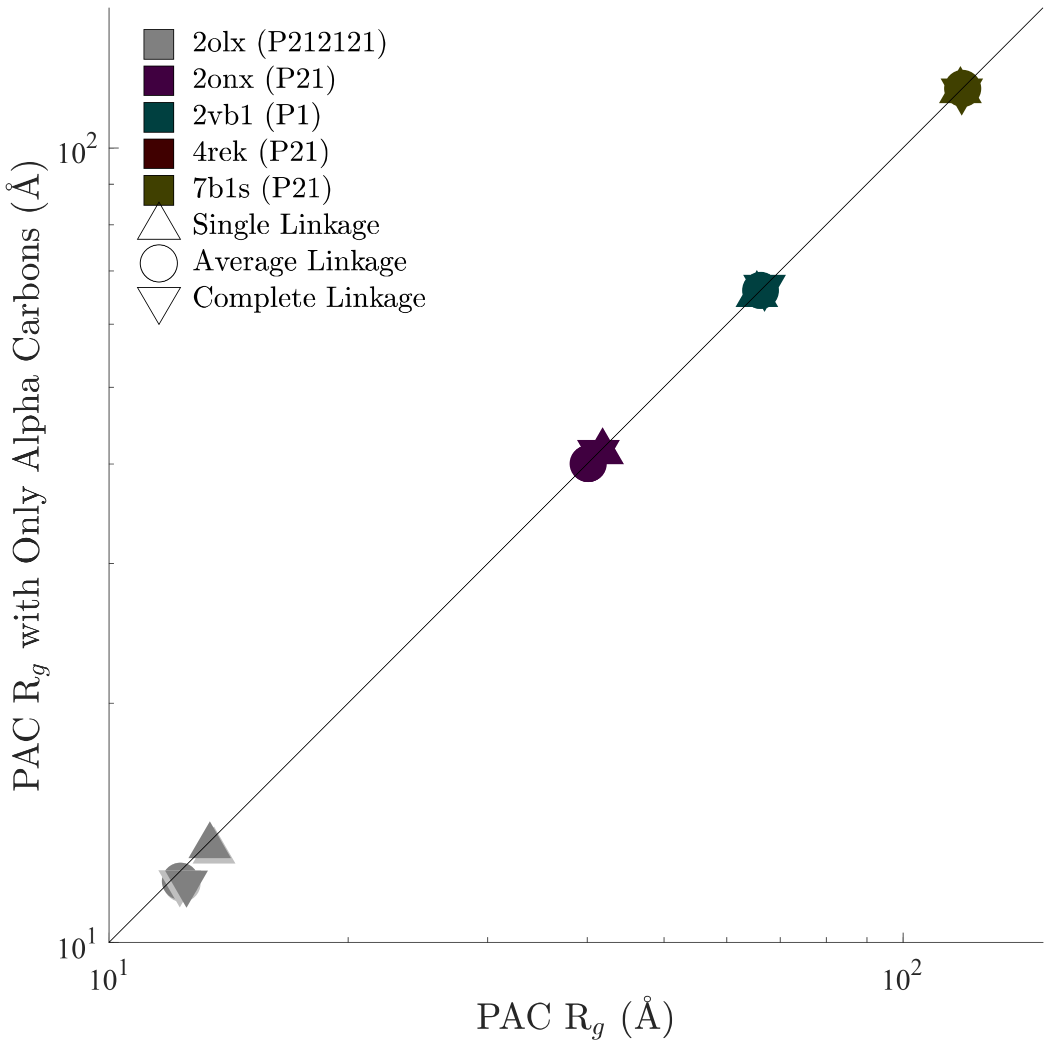

Figure S7. When scaling up to larger systems, the radii of gyration appear to be consistent between single, average, and complete linkages.

Table S1. PAC and COMPACK $RMSD_{20}$ and R_g values for crystal comparisons of the chosen structures for this study.

CCDC ID	COMPACK		Single		Average		Complete	
	$RMSD_{20}$ (Å)	R_g (Å)	$RMSD_{20}$ (Å)	R_g (Å)	$RMSD_{20}$ (Å)	R_g (Å)	$RMSD_{20}$ (Å)	R_g (Å)
ACEMID	0.204	5.775	0.200	5.746	0.225	5.825	0.225	5.833
ACEMID01	0.274	6.127	0.265	6.125	0.341	5.824	0.362	6.120
CBMZPN01	0.367	9.123	0.376	9.149	0.363	9.065	0.372	9.007
CBMZPN02	0.223	9.162	0.228	9.187	0.226	9.074	0.235	9.015
CBMZPN03	0.285	9.482	0.305	9.398	0.302	9.278	0.293	9.149
CBMZPN11	0.150	9.209	0.162	9.155	0.162	9.090	0.171	9.094
CBMZPN12	0.385	9.191	0.377	8.963	0.393	9.112	0.394	8.966
CBMZPN16	0.396	9.093	0.396	9.157	0.398	9.005	0.402	8.957
XAFPAY	0.655	13.738	0.677	13.532	0.782	11.612	0.931	12.599
XAFPAY01	0.219	13.741	0.228	13.462	0.217	11.442	0.213	12.165
XAFPAY02	0.333	13.885	0.322	13.838	0.334	11.485	0.346	12.361
XAFPAY03	0.215	14.263	0.217	12.646	0.208	11.534	0.204	12.552
XAFPAY04	0.296	13.739	0.297	13.781	0.342	11.472	0.356	12.076
2olx	0.926	14.749	0.915	13.563	0.895	12.362	0.921	12.283
2onx	0.979	13.518	0.944	13.395	0.963	12.301	0.952	12.520
YIGPIO02	0.683	14.950	0.685	14.931	0.743	14.170	0.823	14.888
YIGPIO03	0.352	16.234	0.353	16.239	0.348	14.512	0.368	15.326
2vb1			1.019	41.826	1.016	40.135	1.017	41.338
4rek			0.845	65.410	0.856	66.135	0.845	66.876
7b1s			0.992	118.021	0.994	118.841	0.995	118.551
2olx (α)			0.965	13.030	0.927	11.866	0.966	11.889
2onx (α)			0.988	13.229	0.944	11.938	0.965	11.875
2vb1 (α)			0.812	41.428	0.807	40.054	0.812	41.375
4rek (α)			0.693	65.312	0.685	66.164	0.693	66.787
7b1s (α)			0.841	117.958	0.843	118.764	0.841	117.958

Table S2. BCSP comparison values for all submitted structures that were comparable to experimental polymorph B of XAFPAY at 25% bond and 25° angle tolerances. The comparison shaded in orange corresponds to a submitted crystal with vastly increased unit cell size. Single linkage happened to utilize portions of the crystals that do not align well which better represents the similarity between this structure and experiment more so than the similar portions of the crystals alone. As described in the discussion, comparisons featuring crystals with large differences in unit cell volume will benefit from including more molecules (*i.e.*, larger $RMSD_X$)

Submission:<br>Team (Rank, List)	Single Linkage			Complete Linkage		
	RANK	$RMSD_{20}$ (Å)	R_g (Å)	RANK	$RMSD_{20}$ (Å)	R_g (Å)
Neuman, Kendrick, Leusen (R26, L2)	42	2.150	13.83	1	0.391	12.07
Neuman, Kendrick, Leusen (R04, L2)	1	0.244	14.01	2	0.408	12.08
Neuman, Kendrick, Leusen (R02, L1)	1	0.244	14.01	2	0.408	12.08
Price *et al*. (R05, L1)	3	0.288	14.07	4	0.414	12.10
Tkatchenko *et al*. (Price) (R05, L2)	5	0.352	12.16	6	0.550	12.15
Tkatchenko *et al*. (Price) (R02, L1)	5	0.352	12.16	6	0.550	12.15
Brandenbu*Rg* & Grimme (Price) (R04, L2)	7	0.352	13.99	12	0.588	12.03
Brandenbu*Rg* & Grimme (Price) (R08, L2)	4	0.350	14.34	10	0.585	12.01
Price *et al*. (R02, L2)	10	0.398	11.84	8	0.551	12.18
Price *et al*. (R01, L1)	9	0.396	11.84	5	0.546	12.18
Brandenburg & Grimme (Price) (R02, L2)	8	0.393	13.28	18	0.666	12.11
Brandenburg & Grimme (Price) (R03, L2)	17	0.441	12.12	16	0.651	12.02
Brandenburg & Grimme (Price) (R01, L2)	11	0.411	13.99	24	0.714	12.03
Brandenburg & Grimme (Price) (R26, L1)	12	0.415	13.99	14	0.642	12.00
Brandenburg & Grimme (Price) (R31, L1)	13	0.424	14.03	17	0.666	11.99
Brandenburg & Grimme (Price) (R06, L2)	16	0.435	13.29	28	0.734	12.12
Brandenburg & Grimme (Price) (R37, L1)	28	0.494	12.12	21	0.698	11.99
Brandenburg & Grimme (Price) (R38, L1)	29	0.495	12.12	19	0.679	11.99
Brandenburg & Grimme (Price) (R45, L1)	32	0.502	12.12	22	0.706	11.99
Brandenburg & Grimme (Price) (R07, L2)	19	0.450	13.28	30	0.743	12.04
Brandenburg & Grimme (Price) (R39, L1)	18	0.442	14.03	23	0.712	11.99
Brandenburg & Grimme (Price) (R05, L2)	14	0.432	13.99	31	0.750	12.04
Brandenburg & Grimme (Price) (R57, L1)	33	0.509	12.12	25	0.717	12.00
Brandenburg & Grimme (Price) (R34, L1)	34	0.513	12.12	32	0.773	12.08
Brandenburg & Grimme (Price) (R36, L1)	35	0.516	12.12	33	0.775	12.08
Brandenburg & Grimme (Price) (R32, L1)	36	0.518	12.12	34	0.781	12.08
Brandenburg & Grimme (Price) (R46, L1)	37	0.520	12.12	35	0.788	12.08
Brandenburg & Grimme (Price) (R61, L1)	20	0.455	14.02	36	0.792	12.09
Brandenburg & Grimme (Price) (R47, L1)	24	0.486	13.30	26	0.726	12.00
Brandenburg & Grimme (Price) (R59, L1)	26	0.492	12.73	29	0.738	12.00
Brandenburg & Grimme (Price) (R56, L1)	25	0.488	13.30	27	0.734	12.00
van Eijck (R20, L1)	21	0.463	13.52	13	0.592	12.21
Brandenburg & Grimme (Price) (R52, L1)	31	0.502	12.74	38	0.799	12.08
Elking & Fusti-Molnar (R78, L1)	22	0.473	13.52	11	0.586	12.14
Brandenburg & Grimme (Price) (R42, L1)	30	0.499	13.30	39	0.808	12.08
Brandenburg & Grimme (Price) (R44, L1)	38	0.547	12.12	40	0.820	12.08
Pantelides, Adjiman *et al*. (R21, L1)	23	0.483	12.51	15	0.646	12.22
Obata & Goto (R13, L1)	27	0.494	14.11	20	0.682	12.33
Brandenburg & Grimme (Price) (R11, L1)	15	0.434	13.94	9	0.568	12.01
Day *et al*. (R75, L2)	39	0.637	13.53	37	0.797	12.26
Pantelides, Adjiman *et al*. (R13, L1)	40	0.644	12.95	41	0.924	12.24
Mohamed (R88, L1)	41	0.721	14.09	42	1.069	12.41
Average Values	-	0.496	13.12	-	0.678	12.09

Table S3. PAC and COMPACK $RMSD_{20}$ timings for the fastest single comparison of many.

	COMPACK	Single	Average	Complete
CCDC ID	Time (s)	Time (s)	Time (s)	Time (s)
ACEMID	0.019	0.001	0.001	0.001
ACEMID01	0.049	0.002	0.002	0.002
CBMZPN01	0.043	0.003	0.002	0.003
CBMZPN02	0.043	0.003	0.002	0.003
CBMZPN03	0.041	0.005	0.003	0.006
CBMZPN11	0.228	0.007	0.004	0.008
CBMZPN12	0.042	0.004	0.002	0.004
CBMZPN16	0.040	0.003	0.002	0.003
XAFPAY	0.055	0.005	0.002	0.005
XAFPAY01	0.056	0.005	0.002	0.005
XAFPAY02	0.124	0.009	0.004	0.009
XAFPAY03	0.055	0.005	0.002	0.005
XAFPAY04	0.115	0.010	0.004	0.009
2olx	0.160	0.006	0.002	0.005
2onx	0.135	0.006	0.002	0.005
YIGPIO02	0.222	0.010	0.002	0.008
YIGPIO03	0.283	0.010	0.002	0.008
2vb1		2.474	0.047	2.617
4rek		57.042	0.198	57.003
7b1s		1162.476	0.929	1335.136

Table S4. Comparison timings for increasing shell sizes of carbamazepine polymorphs ($RMSD_x$).

	CCDC ID	CBMZPN01	CBMZPN02	CBMZPN03	CBMZPN11	CBMZPN12	CBMZPN16
COMPACK	RMSD20 Time (s)	0.043	0.043	0.041	0.228	0.042	0.040
	RMSD40 Time (s)	0.101	0.103	0.090	0.486	0.095	0.095
	RMSD80 Time (s)	0.185	0.186	0.161	0.829	0.174	0.168
Single	RMSD20 Time (s)	0.003	0.003	0.005	0.007	0.004	0.003
	RMSD40 Time (s)	0.006	0.005	0.007	0.022	0.006	0.007
	RMSD80 Time (s)	0.012	0.011	0.020	0.038	0.012	0.013
Average	RMSD20 Time (s)	0.002	0.002	0.003	0.004	0.002	0.002
	RMSD40 Time (s)	0.003	0.003	0.004	0.012	0.003	0.004
	RMSD80 Time (s)	0.007	0.006	0.012	0.022	0.007	0.008
Complete	RMSD20 Time (s)	0.003	0.003	0.006	0.008	0.004	0.003
	RMSD40 Time (s)	0.006	0.006	0.007	0.023	0.006	0.007
	RMSD80 Time (s)	0.012	0.110	0.021	0.040	0.012	0.014

Table S5. Explicit values of the parallelized computation timings for the ritonavir comparisons.

Number of Nodes	CCDC ID	
	YIGPIO02 Time (min)	YIGPIO03 Time (min)
1	178.8	168.2
2	90.3	85.9
4	45.9	42.8
8	23.8	22.2
16	13.3	12.5
32	7.6	7.4
64	5.2	5.0

Table S6. The increase in the R_g between each method was also evaluated by observing a series of crystals containing molecules with an increasing number of methyl groups between two acetamides. As the molecules get larger the R_g increases, however for COMPACK and single linkage the R_g increases much faster than average linkage due to the selection of molecules included in the comparison cluster.

CCDC ID	Number of methyl groups	COMPACK		Single		Average		Complete	
		$RMSD_{20}$ (Å)	R_g (Å)	$RMSD_{20}$ (Å)	R_g (Å)	$RMSD_{20}$ (Å)	R_g (Å)	$RMSD_{20}$ (Å)	R_g (Å)
JOFHIV00	1	0.490	7.87	0.502	7.86	0.516	7.87	0.497	8.00
ABAWEG00	2	0.380	8.83	0.377	9.01	0.444	7.98	0.587	8.57
ABAWEG01	2	0.505	9.17	0.558	8.96	0.601	7.97	0.634	8.32
ABAWIK00	3	0.254	9.36	0.254	9.36	0.281	8.59	0.311	8.87
ABAWOQ00	4	0.603	10.94	0.604	10.92	0.799	9.12	0.835	9.47
ABAWUW00	5	0.214	10.96	0.212	10.98	0.235	9.50	0.250	9.77
DAMEDA00	6	0.475	13.16	0.480	12.96	0.601	9.90	0.674	10.93
